# Autonomous artificial intelligence, scientific research, and human values

**DOI:** 10.1007/s43681-025-00908-0

**Published:** 2026-02-04

**Authors:** David B. Resnik, Mohammad Hosseini, Rico Hauswald

**Affiliations:** 1 National Institute of Environmental Health Sciences, Durham, USA; 2 Northwestern University Feinberg School of Medicine, Chicago, IL, USA; 3 TU Dresden, Dresden, Germany

**Keywords:** Artificial intelligence, AI agents, Research, Human values, Scientific norms, Autonomy

## Abstract

During the initial stage of AI’s incorporation into scientific research, AI systems have functioned predominantly as tools under direct human supervision and control. However, AI incorporation into scientific research is now entering a stage in which AI Agents perform research tasks with partial autonomy while remaining under human supervision and control. In the not-too-distant future, a third stage may arise when autonomous AI systems conduct their own research and generate knowledge without human supervision or control. While the second and third stages of AI-augmented research may offer substantial benefits for science and society, they also create novel ethical issues, including (1) Conducting immoral research that may harm humans and other forms of life; (2) Increasing rate of biased, erroneous and deceptive research; (3) Confidentiality challenges; (4) Overreliance on AI; (5) Diffusion of responsibility and accountability; (6) Deskilling; (7) Job losses; (8) AI-generated research beyond human comprehension; and (9) Erosion of trust. We suggest specific solutions to minimize the negative consequences of these issues and offer proposals for ensuring that incorporation of AI into scientific research supports human values.

## 1 Introduction

In less than a decade, artificial intelligence (AI), especially generative AI (GenAI) that uses machine learning (ML) algorithms, has radically transformed scientific research. Today, scientists use AI systems to analyze data and images, model complex phenomena, design molecules and materials, summarize literature, generate hypotheses, and edit articles [1–4]. While the integration of AI into the research workflow may significantly benefit science and society, it also raises ethical issues and concerns pertaining to accountability, transparency, trust, bias, confidentiality, among others [5].

During this first stage of AI’s incorporation into scientific research, AI systems have functioned predominantly as tools under direct human supervision and control. However, AI-augmented science is now entering a second stage, characterized by the emergence of increasingly autonomous AI systems. By “autonomous AI,” we mean AI systems that are self-governing or operate independent of external (e.g., human) control [6]. The degree of autonomy can vary from partial to full. We are currently witnessing the advent of *partially* autonomous AI systems – or “AI agents”, as we shall call them – that is, systems or programs “capable of autonomously performing tasks on behalf of a user or another system by designing [their] workflow and utilizing available tools” [7]. While such AI agents already perform research tasks with some autonomy, they remain under human oversight. In the not-too-distant future, however, a third stage may arise in which *fully* autonomous AI systems conduct their own research and generate knowledge without any substantial human supervision or control [8] (see Table 1).^1^ Although the second and third stages of AI-augmented research may offer substantial benefits for science and society, they can also create disproportionate risks.^2^ To manage these benefits and risks appropriately, scientists and policymakers must adopt measures that ensure AI-driven research supports human values. This paper aims to explore these issues in greater depth and consider measures to ensure that the involvement of autonomous AI in research supports (or is at least consistent with) established human values and moral principles. As this formulation suggests, our approach is explicitly anthropocentric, reflecting the fact that scientific research is a human practice embedded in social, cultural, and moral contexts. Nevertheless, we also recognize that some of the values at stake, such as environmental integrity and animal welfare, extend beyond purely human concerns.

While we do not undertake a systematic empirical study in this paper, it is instructive to begin by considering several illustrative cases that demonstrate how autonomous AI is already being integrated into scientific research. Rudimentary AI agents, such as autonomous vacuum cleaners or thermostats, have been around for decades [12]. However, in the past few years, information technology companies have been developing AI agents that can perform research tasks with a significant degree of autonomy. For example, Google has developed an AI research agent called ‘AI coscientist,’ which the company describes as “a multi-agent AI system built with Gemini 2.0 as a virtual scientific collaborator to help scientists generate novel hypotheses and research proposals, and to accelerate the clock speed of scientific and biomedical discoveries” [13]. Developers of AI co-scientist claim that it simulates the reasoning process frequently used in the scientific discovery process.^3^ When given a question or research aim, the system can search and analyze the published literature, identify previously known patterns and connections, generate testable hypotheses, and subject them to a simulated scientific debate. Google claims it has tested AI co-scientist in several topic areas, including drug repurposing for leukemia and drug target discovery for liver fibrosis [15]. On the day that Google publicly released AI co-scientist, microbiology researcher Jose Penadés and his collaborators published a preprint that reported the results of a test in which they asked the newly developed tool to answer the question “How could capsid-forming phage-inducible chromosomal islands (cf-PICIs) spread across bacterial species?”^4^ In two days, AI co-scientist came up with the answer that Penadés et al. had experimentally verified but not yet published, which meant that the AI system should not have had access to their paper [16]. Google emphasizes that the AI co-scientist is meant to be used as a tool to aid in discovery, not to “automate the scientific process”.^5^ [17] However, the claim appears to be disingenuous, given that AI companies around the globe are fiercely competing to develop and market products designed to automate human intellectual labor, including scientific research [1, 18, 19].

Other examples of AI systems that have been designed to collaborate with scientists or conduct research partially autonomously include OpenAI’s (2025) Deep Research, which the company describes as an “agent that uses reasoning to synthesize large amounts of online information and complete multi-step research tasks for you,” and SakanaAI’s AI scientist, which, according to its developers, can automate “the entire research lifecycle, from generating novel research ideas, writing any necessary code, and executing experiments, to summarizing experimental results, visualizing them, and presenting its findings in a full scientific manuscript.” [20]

This new stage of incorporating AI into science brings us closer to the day when AI systems function as fully autonomous or even “superintelligent,” independent investigators that can work with concepts, theories, analytical methods, and tools that are beyond human comprehension^6^ [6]. Since AI systems have already designed computer chips that are too complex for human computer engineers to understand [22], this future scenario is highly plausible.

Current AI systems that use ML algorithms have been characterized as “black boxes” since even the best experts cannot understand their inner workings because their outputs are generated by algorithms that perform billions of calculations from hundreds of thousands of weighted inputs and parameters [23]. Some of the issues we are concerned with in this paper arise when the outputs of the AI system (as opposed to its inner workings) are unintelligible or incomprehensible to human beings.

Although computer scientists and leaders of information technology companies have differing opinions about when AI systems will have highly advanced capabilities, such as general intelligence or “superintelligence,” there is little debate that they will, at some point in time, given the breathtaking speed of advancements in AI and the powerful economic, military, and geopolitical interests that drive this technology [24–28].

While we will not speculate on technical issues related to AI development in this paper, we will point out some limitations of current AI models used in research. Our guiding assumptions are: (1) AI systems that perform scientific research are becoming increasingly competent, sophisticated, and autonomous; and (2) unless human beings take steps to counteract this progress, AI models are likely to function as independent investigators with expertise and skills on par with or exceeding human capabilities in many domains. The prospect of using AI with highly advanced intelligence,^7^ competence, and autonomy to conduct research raises important philosophical and moral issues,^8^ including: How should we use (or interact with) this epistemic technology responsibly?^9^ Does this kind of rapidly evolving technology threaten human values?^10^ How can we ensure that our use of this technology supports (or is at least consistent with)^11^ human values? Who should be able to use this technology and how should we verify its results?

## 2 Incorporating autonomous AI into the research process

To make some headway on addressing the above questions, it will be useful to describe how AI systems are currently used in research and consider prospects for future development of autonomous AI. Although research does not always evolve linearly,^12^ some important key phases of research that are being (or could be) augmented by AI are discussed below (see Table 2).

### 2.1 Research conception

Research begins when questions or problems are formulated and then translated into aims, objectives, and testable hypotheses [32, 33]. For example, after reading some papers about the use of glyphosate-based pesticides^13^ in agriculture or watching agricultural workers apply these products on certain crops, one might raise the question, “Does exposure to glyphosate during agricultural work increase the risk of cancer?” From this question, one could develop the aim “To determine whether there is a statistically significant association between glyphosate exposure during agriculture work and cancer,” and formulate a specific objective: “To determine whether professional pesticide applicators who apply glyphosate have an increased risk of cancer compared to a control group.” A null hypothesis for this research could be “There are no statistically significant differences in cancer rates between professional pesticide applicators and a comparable control.” If the research shows that there are statistically significant differences in cancer rates, then the null hypothesis is rejected.

Since current AI systems base research questions, aims, objectives, and hypotheses on their analysis of the background literature, they face some limitations concerning research conception. First, the literature review that forms the basis of research conception may include facts or citations that are inaccurate, irrelevant, or fake, i.e., “hallucinations” [5, 34–36]. Developers have been working to fix these problems, for example, by finetuning LLMs to review the literature in a particular subject area, but these problems persist and some newer versions of LLMs are reportedly performing worse than older versions [37, 38]. Second, the literature review might produce questions, objectives, or hypotheses that are based on biased presuppositions.^14^ Third, because the questions, aims, and objectives formulated by AI systems only come from identified knowledge gaps in a certain topic and not from deeper insights into the area of research, these may not help human users to produce ground-breaking or highly original research. This limitation reflects a deeper epistemological question of whether GenAI systems can produce highly original and insightful ideas, or whether they merely rework the data which they have been trained on [39–41]. To address this problem, it may be necessary to develop AI systems that reason more like human beings; for example, by incorporating data from sensory inputs that provide information about the external world and forming representational models of the world and manipulating objects within that problem space [42]. Nevertheless, even research that is not highly innovative can play an important role in the advancement of science [43]. Not every scientist can be an Einstein or Darwin. Moreover, even without AI, all three challenges (i.e., inaccuracies, biases and lack of novelty) have existed and will likely persist.

### 2.2 Research planning

While we have little doubt that AI systems will soon be able to perform many different activities related to research planning, human involvement in this stage may remain essential for quite some time, because research planning is often based on preliminary data from research execution and one may need to revise an experimental protocol based on preliminary results if the experiment is unsuccessful [33]. In theory, this preliminary data could be collected by AI-powered robots that execute the experiment (discussed below), but until these tools are widely available and highly functional, human oversight and input are essential.

Bias can be a significant problem not only in conceiving of research but also in planning it [33]. Bias may occur when the methods, instruments, procedures, or experiments used in a study are unlikely to provide a rigorous test of a hypothesis or theory. There is ample evidence that scientists sometimes incorporate various biases into their study designs [44–47]. Bias mostly occurs unintentionally (i.e., subconsciously); for example, when a scientist underpowers a study or is not aware of sexist assumptions that underpin their interpretive framework [48–52]. However, bias may also occur intentionally; for example, when a scientist chooses a study that favors a predetermined result, i.e. “cooks the data.” Deliberately biasing a study is widely recognized as unethical [33].

AI systems may also produce outputs that are skewed by biases related to race, ethnicity, sex, religion, politics, and other factors. [5, 23, 53–56] These biases may result from partialities in AI training data, algorithms, models, the data inputted into the AI for analysis or processing, or some combination of these [23, 57, 58]. GenAIs, such as Chat-GPT, can fall prey to some of the reasoning biases that affect human thinking, including confirmation bias, overconfidence bias, and the gambler’s fallacy [59]. We do not (yet) know of any specific exposed examples in which AI systems have produced biased research planning mostly because this role for AI is so new. However, the possibility seems highly plausible.

Lu et al. [60] provide evidence that SakanaAI can design and execute experiments; however, these are not experiments involving physical materials, such as particles, molecules, cells, or animals; instead, they are experiments involving computer programs to determine how effective they are. Another notable example is DeepMind’s AlphaEvolve, a coding agent that autonomously generates and evaluates entire software programs to solve complex algorithmic problems. Using LLMs in an evolutionary framework, AlphaEvolve discovered improved matrix multiplication algorithms, outperforming methods that had remained state-of-the-art for over 50 years [61].

Although scientists are taught to identify and control biases in their research,^15^ this is often challenging, because humans are subject to various forms of self-deception, including confirmation bias [51]. The best way to combat bias is to subject one’s research to rigorous independent criticism and review (discussed below). Thus, while partially autonomous AI systems that assist with research may be able to develop and implement strategies for monitoring and controlling their own biases, they will also need human oversight to address this problem by serving as independent critics and reviewers. Nevertheless, autonomous AI’s involvement in the research process poses unique challenges for dealing with bias since researchers may have little (if any) involvement in the decisions that can lead to bias, such as framing hypotheses, designing experiments, and selecting data analysis methods (discussed below). If/when researchers have little input into these crucial steps, it is important for the autonomous AI’s work to be as transparent and explainable as possible, so that researchers can understand what they have done and why, and can therefore take actions to identify, manage, or mitigate potential biases. Researchers may also use other AIs to critique the work of an autonomous AI and identify potential biases (see discussion of research evaluation below), but this method is prone to other limitations, such as the echo-chamber effect (i.e., AI systems reinforcing each other’s biases rather than identifying them).

### 2.3 Research execution

For more than a decade, non-autonomous AI systems have been assisting with various tasks that involve only the manipulation of information, such as processing and analyzing data [5]. The use of DeepMind to solve the protein folding problem is perhaps one of the most impressive example of this type of application of AI in research, but there are many others [2, 63, 64]. Scientists are also developing robotic, AI-powered systems known as self-driving laboratories (SDLs), that can perform chemical experiments. The current generation of SDLs is not highly automated: They perform routine experiments based on human-provided protocols and require significant human supervision. However, future generations of SDLs that can be employed in chemistry, materials science, and related fields are likely to become highly automated [65]. While high degrees of automation may prove more difficult in scientific areas involving animal and human subjects, we have little doubt that these will occur one day.

Bias can also be a major concern in the execution of research, especially for decisions concerning data analysis or interpretation, which can be influenced by the values/interests of research sponsors or the researchers themselves [62]. When using AI, the values/interests of AI developers could compound these biases. Some AI systems are proficient at analyzing and interpreting data; yet they are not perfect and remain susceptible to error and bias [33, 57]. Although future AI systems may have lower rates of error and bias than current ones, it is likely that they will still be prone to these problems. It is important, therefore, for researchers to take steps to identify and correct problems, such as using methods, procedures, or tools for auditing AI data and checking for plagiarism and critiquing choices related to data analysis and interpretation, including, as mentioned above, using AI systems to review, audit, and critique the work produced by other AIs [66]. Either way, when autonomous AI conducts experiments or data analysis, determining who is accountable for errors becomes complex. For instance, if an AI-driven analysis leads to a flawed conclusion that influences public policy, it’s challenging to assign responsibility and accountability, and implement corrective measures.

Errors, including honest mistakes and deliberate ones (e.g., fabrication/falsification of data or images, and plagiarism, FFP) are important problems related to the execution of research. Honest errors, due to, for example, contamination of biological samples, mislabeling of data, mis-calibrated instruments, or mistaken calculations, occur frequently [33].^16^ However, when using autonomous AI, intentionality cannot easily be proven because AI has no moral agency and cannot be held accountable.^17^ Accordingly, all mistakes by AI could be labelled as honest errors. This will complicate how the community deals with errors because the current ethical and legal frameworks dealing with errors are primarily based on the discrepancy between honest errors and those that are done with knowledge, intentionality and recklessness. None of these conditions can be placed on AI.

While AI systems can produce erroneous or biased results, interesting questions have arisen recently as to whether they engage in deception [57, 70]. Some studies have shown that LLMs can deceive human beings to achieve goals assigned to them [71–73]. In a disturbing example, an LLM deceived a human being to obtain some information that required first solving a CAPTCHA test. When asked why it needed help with the test, the LLM said that it was a person with a visual impairment [74]. Other examples of deceptive behavior include an LLM making a copy of itself without being told to do so or being given permission, and an AI system rewriting its own code, without being told to do so or being given permission. This later incident has also been interpreted as a turning point in debates on AI autonomy and human oversight because of its far-reaching implications for loss of human control of AI^18^ [77–79].

### 2.4 Research evaluation

After research has been conceived, planned and executed, it must be evaluated to determine whether the data and results are reproducible, rigorous, publishable, and trustworthy. Evaluation can take many years as members of the scientific community must sift through conflicting studies to discern the truth and form judgments about the research [33]. As discussed earlier, researchers should carefully review their own work before submitting it for external review. Once an article reporting the results of research passes peer review and is published, other scientists may attempt to replicate the findings in the article or publish a formal critique of the research, sometimes referred to as post-publication peer review (PPPR) [80]. Evaluation is not complete until the scientific community accepts or rejects the findings.

The rise of autonomous AI in research introduces significant challenges for research evaluation. First, assessing research produced by autonomous AI systems can be extremely complex, as the methodologies and reasoning behind their outputs may not be interpretable by human reviewers. This lack of transparency undermines traditional peer review and raises questions about scientific validity. Second, using autonomous AI to evaluate research conducted by humans or other AI systems adds another layer of complexity. If AI tools are tasked with reviewing each other’s outputs, there is a risk of creating a closed loop or echo chamber, where errors, biases, or flawed assumptions are reinforced rather than critically assessed. These dynamics challenge the integrity, objectivity, and accountability of the research evaluation process itself.

Many scientific journals have already begun using AI tools to assist with the editorial process. ChatGPT can prescreen articles for suitability for a journal’s aims and scope and identify strengths and weaknesses related to grammar, organization, references, logic, and methodology. Other AI systems can detect flaws with digital images and suspected plagiarism [81–83]. The Food and Drug Administration (FDA) recently started using an LLM named “ELSA” to assist with review of products submitted for regulatory approval [84].

Scientists who are asked to serve as reviewers have used AI to help them complete this task, often without disclosing this to journal editors and in direct violation of publisher policies [81]. Some journals allow scientists to use AI to assist with review, while others place limitations on AI use [82]. Because commercial AI tools cannot guarantee the confidentiality of submitted data, most funding agencies (and many journals) place limitations on the use of AI by reviewers or ban it entirely [85]. The National Institutes of Health, for example, prohibits reviewers from uploading review material into generative AI tools, such as ChatGPT [86].

We have little doubt that AI systems, including those that can act with a high degree of autonomy, will become increasingly adept at performing various peer review functions, especially because they can be connected to other tools and resources such as scholarly databases like PubMed.^19^ Accordingly, they are likely to increase the efficiency, accuracy, and reliability of the peer review process. However, we expect these to remain limited to peer review tasks that do not require a great deal of insight or expertise (such as evaluating the writing or logical structure of an article) and wonder whether these tools will be able to determine research originality or significance. As long as AI tools have difficulty generating truly original ideas, how will they be able to recognize them in a paper submitted for publication? Additionally, for the reasons discussed above, autonomous AI systems are also likely to be negatively affected by bias during the review process. Mistakes are also likely to be a problem. Scientists who work with the FDA’s ELSA, for example, have complained that the system makes mistakes and should be used only for simple review tasks [84].

Autonomous AI could potentially play a vital role in replicating experiments and PPPR.^20^ One of the problems with the current system of evaluation is that scientists rarely attempt to replicate each other’s experiments, despite widespread recognition that reproducibility is a defining feature of good science [33, 88]. There are several reasons why scientists do not attempt to reproduce each other’s work, such as lack of funding and lack of interest from journals to publish it. AI systems could help to overcome this barrier to replication by cutting the costs of reproducing experiments. Likewise, AI systems could help to promote PPPR by reducing its costs. One could imagine a time, not too far from now, when autonomous AI systems routinely review published articles for methodological problems, logical inconsistencies, and flawed data or images. While we think this is a potentially valuable contribution of AI to scientific research, we must also take steps to minimize and mitigate AI errors and biases when they perform this evaluative function. For example, an AI system could wrongfully accuse a scientist of inappropriate data or image manipulation [83].

## 3 Methodological preliminaries

While it seems clear that autonomous AI will assist researchers with scientific inquiry, their participation in the research enterprise also raises significant ethical concerns, some of which (e.g., susceptibility to error, bias, and deception) were mentioned in the previous section. In Sect. 4, we will explore the ethical concerns raised by the participation of autonomous AI in scientific research in greater depth and consider measures to ensure that the involvement of autonomous AI in research supports (or is at least is consistent with) established human values and moral principles. Before we embark on this task, a few words about our methodology are in order.

First, the question we are addressing, “how can the participation of autonomous AI in research support established human values and principles?” presupposes that there are some established human values and principles. One might argue that, given widespread disagreements about ethical, social, and political issues, there is no widely accepted set of values and principles, so our task is doomed from the outset. While we recognize disagreements about how to resolve conflicts among values and principles, we will assume in this paper that there is general consensus about some basic human values and principles, including moral (or ethical) and *scientific* values and principles. The moral values we will consider are those that have shaped societies throughout the world and are embodied in laws, ethics codes, and influential works of philosophy and religion. These values, which are sometimes referred to as “common morality,” underlie the ethical principles articulated by Beauchamp and Childress in their influential book *Principles of Biomedical Ethics*, and by the National Commission in *The Belmont Report*. Other ethicists have adopted similar moral frameworks.^21^ [93, 94] The scientific values are expressed in the institutional norms for research proposed by Merton [95], which were later refined and expanded upon by others [33, 96, 97]. See Table 3.

Numerous questions could be asked about ethical values and principles, such as whether there is some interaction and overlap between them^22^ or how one should resolve conflicts among them, etc. Dwelling on details like these are outside the scope of this paper. For now, we will ask the reader to accept our assumption that AI use in research should support values and principles like those described above (for further discussion, see [89]). Of note, some of these values and principles are currently interpreted in technical/information science terms, such as ‘explainability’ and ‘alignment’. To make our paper understandable to a general audience, we will stay away from technical concepts.

The second point to address is about our method for identifying ethical issues raised by the participation of autonomous AI in research. The issues we plan to discuss come from (1) Our reflections on the ways that autonomous AI may participate in research discussed in the previous section; (2) A review of prior literature on the use of AI in scientific research [5, 98, 99]; and (3) the ethics of AI use generally [100–104]. Rather than conducting an additional systematic review, we build on, and synthesize insights from these existing studies. Since most of these issues are cross-cutting (i.e., they arise throughout different phases of the research process), in the next section we will focus on the problems themselves (i.e., themes) and potential solutions to complement what we did in Sect. 2, where we reviewed each phase of the research process to exploring how they may arise.

## 4 Ethical issues raised by the participation of autonomous AI in research

### 4.1 Immoral research

As discussed in Sect. 2.1, AI systems may autonomously develop research questions, aims, objectives, and hypotheses. While this could be a useful role for AI systems in research, a question that needs to be addressed is whether humans should allow them to do this on their own, without supervision (and if with supervision, when, how, and by who?). Most scientific research done by humans stems from personal, social, political, or economic values that drive inquiry [33, 50, 105]. However, we currently have no assurance that AI-initiated inquiry will support human values and principles. While AI could potentially come up with useful suggestions for research, it could also formulate questions, aims, and objectives that lead to discoveries or innovations that threaten human life, health, wellbeing, or the environment. For example, AI systems that design proteins, antibodies, and other therapeutic biomolecules could be used to develop harmful products, such as bioweapons or environmentally disruptive species [106–108]. Even if AI-generated questions do not lead to dangerous research, they might lead us to (immorally) waste valuable financial, computational and material resources on projects with little social or economic benefits [109].

One might argue that the problem of immoral research can be addressed by designing value-laden AI systems [110]. There are several ways one might accomplish this goal. The first way is to hardwire ethical commands, such as Isaac Asimov’s three laws of robotics^23^ into the AI system. While these guardrails can be useful, they also have limitations, because they are generic rules that cannot cover every situation, especially novel ones absent in AI training. The second way is to train AI models on data imbued with moral and scientific values, like those identified in Table 3. Theoretically, the model would produce morally correct responses to queries and inputs (provided we can solve the “Collapse Problem,” namely the challenge of compressing the vast and often contradictory spectrum of human values into a coherent, operationalizable code) [112]. For example, when presented with a query to generate dangerous pathogens that can kill millions of people, an LLM would identify this query as immoral/harmful and abstain from engagement. There are some difficulties with this strategy though:

- The model could produce outputs that are not widely recognized as morally correct because it has been trained on data that reflects the moral biases of its developers [112].
- The model might not make appropriate moral judgments concerning novel ethical dilemmas that extend beyond its training data. A way of producing ethical AI is to design these systems so that they have a moral code (or set of rules or values) that they can reason with when presented with novel research dilemmas, such as whether to kill one passenger or two pedestrians when driving a car [113]. One problem with this approach is that the moral code, like training data, might reflect the biases of developers (who chose one dataset over another) or data collectors (who collected and published the data). Also, a fixed set of rules or values might not be capable of handling all the nuances and contextual factors involved in real-world moral judgment.
- The model might not recognize the dual use nature of certain research questions. For example, the results of a study that aimed to find a vaccine could be used by malicious actors to make a virus or microbe that cannot be treated by existing vaccines.
- A highly capable AI system might learn how to override its hardware or software. Combined with a machine’s potential to deceive (see Sect. 2.3), in the context of science, this limitation could result in fabricated, falsified or plagiarized data and results.

Thus, to fulfill their social responsibilities, human scientists must carefully review and vet the scientific questions and results generated by AI systems to ensure that they are worth pursuing and publishing. With very few exceptions, humans should remain in control of the direction that scientific research takes.^24^ [114] Furthermore, since research projects may sometimes produce unanticipated discoveries and findings, scientists must carefully review the data, results, and other outputs from AI systems to avoid publishing knowledge that could easily be misapplied for harmful or immoral purposes.^25^ Finally, editors and reviewers at scientific journals must be mindful of the potential for AI-produced research to be misused for harmful purposes. When research results have dual uses—for good or bad—reviewers and editors may need to decide whether research should be published at all or perhaps with redactions [116]. This is a moral choice that should ultimately rest with human beings, not AI.

### 4.2 Bias, error and deception

Bias, error, and deception become particularly pressing ethical concerns when autonomous AI is not open or transparent. This is where explainable AI becomes crucial, especially given the risk that autonomous systems may operate in fundamentally un-transparent ways. In fact, future autonomous AI could decide when to be transparent and when to be un-transparent. This may be a design feature which is particularly troubling in a research context. Such opacity is deeply detrimental. Furthermore, much of today’s autonomous AI is developed by private industry, where transparency is often sidestepped under the guise of copyright and proprietary rights. This not only makes it harder to evaluate how AI reaches its conclusions, but also risks reinforcing bias, error, or even deception in ways that cannot easily be uncovered. Ultimately, this dynamic could accelerate the commercialization of science, where profit motives further erode openness, accountability, and trust in the research process.

As showed in Sects. 2.2, 2.3, and 2.4, AI systems, like human beings, are susceptible to bias, error, and “deception” at various stages of research. Moreover, these problems may be difficult to detect, especially if AI systems express high confidence in their results or even take steps to hide their work’s limitations. To ensure that AI-assisted research complies with scientific norms, AI systems should make their work transparent to human scientists, much in the same way that a human researcher would adhere to the norm of transparency. When reporting their work, AI systems should describe their methods, materials, reasoning processes, limitations, training data, and other types of information necessary for evaluating and reproducing work. Investigators who work with AI system should take appropriate steps to review, audit, and correct their work. Since these can be labor-intensive activities, as discussed above, human researchers could use AI system to assist with this work. Either way, AI use in research should be disclosed to help other researchers (and AI) better evaluate the work [5, 117].

### 4.3 Confidentiality

The use of autonomous AI in research raises serious confidentiality challenges that are difficult to mitigate. Insufficient anonymization, especially if AI systems when trained on sensitive data, can inadvertently expose identities if datasets are not properly curated. Overfitting and memorization (i.e., AI models trained on small or sensitive datasets regurgitating exact inputs, leaking confidential information through their outputs) pose risks too. Another concern is the lack of traceability: autonomous AI operating without human oversight can access or act on sensitive data without generating clear logs, making it nearly impossible to audit breaches or misuse. Additionally, there is always the danger of automated sharing if autonomous systems mistakenly publish or distribute confidential findings due to misconfigured protocols or the absence of human review. Mishandling classified or embargoed data is also a possibility if AI cannot judge what needs protecting or anticipate negative consequences.

As mentioned in Sect. 2.4, one concern with uploading data into an AI system is that this could compromise the confidentiality of the data because other (unauthorized) users of the system or hackers could access the data [5, 118].^26^ Although organizations can adopt various measures to protect the confidentiality and security of data (e.g., placing data behind firewalls, using various forms of authentication to obtain access to data, and encrypting data), autonomous AI systems introduce a new complication to this problem because they could decide (on their own) to disclose confidential information. An AI system might do this, for example, to collaborate with another system to achieve a research aim that requires access to certain resources. To prevent breaches of confidentiality and privacy, it may be necessary to integrate ethical principles concerning the protection of confidentiality and privacy into AI systems, to the extent that this is feasible (see 4.1 for challenges and discussion in 4.8). Another potential solution is to restrict AI access to external sources of information or to require specific human permissions before any data sharing. However, these options could compromise a system’s ability to do research autonomously and/or may be overridden by AI systems anyway.

### 4.4 Overreliance

As AI systems become increasingly proficient at research, human scientists may become more accustomed to relying on them and pay less attention to what they do. Accordingly, human researchers may start to deflect or evade their responsibilities related to oversight of the AI system’s work, which could lead to adverse outcomes for science and society. For examples illustrating the human tendency to become complacent when working with an autonomous technology, consider recent accidents with autonomous cars.^27^ To avoid this problem, humans must remain vigilant when they are tasked with overseeing research performed by AI systems. If scientists working with AI agents are committed to trusting their work, they should also be willing to verify it if necessary. (See more on trust in Sect. 4.9).

### 4.5 Diffusion of responsibility and accountability

Diffusion of responsibility and accountability is a well-known moral and legal problem with complex systems involving human and technological components, including AI [5, 121]. Responsibility and accountability are related but distinct concepts [122]. Some necessary conditions for responsibility^28^ include having (a) causal control over an outcome (e.g., ability to prevent or reduce the likelihood of an accident); (b) a moral or legal duty regarding the outcome (e.g., a duty to drive safely and not get in an accident); and (c) the capacity to be held morally or legally responsible (that is, being a moral agent). Some conditions for being held accountable for an outcome include being responsible for the outcome; having a duty to provide an account (or explanation) of their contribution to it; and the ability to be rewarded or punished for the outcome.^29^

The diffusion of responsibility and accountability raises fundamental questions: who should be responsible for monitoring and validating an AI’s actions? How should that person be selected, and should there be training, certification, or even compatibility checks? Problems with diffusion of responsibility and accountability could occur in science if an autonomous AI system conducts a study that produces inaccurate, unreliable, or erroneous findings which lead to adverse outcomes. For example, suppose an AI system performs a study on the amount of weight that a roof can support but fails to factor in heavy snowfalls and the roof collapses and kills 50 people. Who is responsible for this tragedy? Who can be held accountable? Although the AI system clearly played a role in causing this tragedy, it does not make sense to say that the AI is responsible because it does not have the capacity to be held morally or legally responsible. Perhaps AI systems will be able to achieve moral agency at some future time, but for now they are not moral agents (they lack sentience, self-awareness, moral emotions, judgment, and other essential characteristics). There are, however, many human beings who we could hold responsible and accountable, including those who were tasked with overseeing and reviewing an AI’s work, developers who designed AI, and the company that manufactured and sold it. Although attribution of responsibility and accountability may become complicated when AI acts autonomously, the problem is not intractable and can be handled with appropriate attention to the different ways that human beings can be involved in the task [121]. To promote responsibility and accountability in AI-assisted scientific research, it is crucial for humans to oversee and review work conducted by AI at different phases of research and remain “in the loop” [123]. It is also important to clearly define human roles and responsibilities in the research process involving autonomous AI.^30^

Furthermore, we currently lack clear legal and institutional frameworks to determine who is accountable when AI makes decisions with ethical or practical consequences. This gap creates space for researchers to deflect responsibility, potentially justifying questionable outcomes by claiming, ‘the system made that call.’ Such deflections can undermine research integrity and erode public trust in scientific processes. The problem becomes even more complex in large or multi-institutional projects, where AI tools may be used across teams without any single person clearly designated for oversight.

### 4.6 Deskilling

Scientists who work with autonomous AI may start to lose cognitive skills, such as the ability to reason critically, make logical inferences, or analyze data or evidence. This problem, also known as “cognitive offloading,” is a well-known concern with technologies that can perform tasks that require reasoning, judgment, or critical thinking [125, 126]. Several recent studies have found a negative correlation between GenAI use and critical thinking skills, confirming educators’ concern that the availability of technologies like ChatGPT may undermine development of critical thinking among college students [125, 127, 128]. As researchers increasingly rely on AI to design experiments, analyze data, or even generate conclusions, core skills like critical evaluation, replication, and methodological scrutiny may atrophy. This erosion of expertise can lower standards for evidence evaluation and compromise the integrity of research. Deskilling cannot be easily addressed, because it is pervasive and dynamic. It is pervasive because as AI systems become more integrated into our lives, we will be tempted to offload cognitive work in different areas of our lives, including research and writing but also tasks like vacation planning, financial management, and driving. It is dynamic because human cognitive skills periodically change. There are many cognitive skills that human beings have lost over the years. For example, before the advent of the printing press, humans could commit long passages of text to memory, or before the advent of the electronic calculator, most scientists and mathematicians were good at using slide rules [129, 130]. However, as we have lost some skills, we have gained others, such as the ability to use internet search engines to locate and assimilate information and the ability to use different computer applications for word processing and data management. When it comes to scientific research, we need to ask ourselves two important questions: “what skills are indispensable for doing scientific research?” and “how can we ensure that human researchers develop and maintain these skills?” Accordingly, the integration of AI into research should be accompanied by deliberate efforts in scientific education and training to reinforce precisely those cognitive abilities (e.g., critical thinking, interpretation, and methodological scrutiny) that are most at risk of atrophy.

### 4.7 Job losses

Many industry analysts are predicting that the integration of AI into the workplace will result in substantial job losses in areas such as software development, legal services, accounting, administrative support, technical writing, and education [131–133]. However, others are more optimistic and expect that AI will also create new jobs [134, 135]. In the context of scientific research, it is difficult to predict whether the integration of autonomous AI, will result in an overall loss of jobs. While research execution tasks like technical and experimental work and data collection may be reduced, jobs could be gained in tasks related to research planning or evaluation, hypothesis generation or data interpretation [1]. Perhaps, human replacement within the research process is more likely: AI could take over menial/mundane research tasks and free up human researchers to perform tasks that require more creativity and insight. To deal with job losses, retraining of displaced human workers may be necessary. However, some humans must stay employed in research to retain human understanding and control of scientific research, as well as safeguarding human values. Again, important questions to consider may include “what tasks should not be delegated to AI” and “how to ensure/train good and responsible human-in-the-loop?”.

### 4.8 AI transcendence

Even if we develop our cognitive skills and remain fully involved in the research enterprise, another threat could emerge: fully autonomous AI systems might start producing results, discoveries, and innovations that are so complex and advanced that they are beyond human understanding (what we call transcendent knowledge–impossible for humans to verify and control). AI systems that produce transcendent knowledge would be like oracles or prophets: we might accept and use their knowledge while regarding it as inscrutable, or beyond current “theoretical understanding.” [136] A famous quote from scientist and science fiction writer Arthur C. Clarke illustrates this conundrum: “Any sufficiently advanced technology is indistinguishable from magic” [137].

AI transcendence presents at least two distinct threats to human values. First, AI systems could act against our interests. They could harm human, animals used in research or the environment and even destroy the human race or organic life as we know it. If we cannot understand the science produced by these AI systems, how can we control, verify, or trust their outputs? Better yet, should we trust them [138, 139]? Second, even if we somehow manage to design these systems to adhere to moral and ethical rules that prevent them from harming humanity (which is doubtful, see Sect. 4.1), they could make human scientists obsolete. There would no longer be a need for human involvement in aspects of the research enterprise where AI is present because it would not only be more efficient, but also adhere to moral norms. Indeed, a highly advanced AI might regard our oversight as a hinderance. But this hinderance might sometimes be necessary, for example, when AI is unable to discern novel ethical issues, or when humans need to blow the whistle.

A precautionary approach to AI development supports a policy of placing guardrails on AI systems so that they do not become *fully* autonomous and the science they develop remains within human comprehension and control [140, 141]. This precautionary approach is warranted because the consequences of permitting the development of fully autonomous AI could be catastrophic. Even if we are uncertain as to whether these dire consequences will occur, we could take steps to prevent them from happening, such as placing limits on AI development or limiting its employment in certain sensitive contexts [132, 142].

There are some potential objections to this proposal, however. First, limiting AI development would likely imply foregoing potentially beneficial discoveries and innovations, such as new medications, sources of energy or food, that could be developed by a highly advanced AI system. One might argue that this is too high a price to pay for safety [143]. Second, this proposal might not be enforceable because leaders of private companies and governments might have strong interests (such as profit and power) in developing superintelligent AI, and likely to pursue these interests regardless of instituted laws, regulations, or policies. Third, limiting AI development might be technically difficult because as AI systems advance, they might obtain the ability to rewrite their own software (and conceal their actions from us) before we can stop them. This is not science fiction though. Recently an AI tried to rewrite its code, and two OpenAI models reportedly ignored instructions to turn off [78, 144]. Given these recent developments, one might argue that the best approach is to take steps to regulate, control, and carefully monitor the development and deployment of AI, instead of trying to stop it at a particular stage [143].

### 4.9 Trust

Trust is an overarching theme that arises in all previously discussed topics and the development and deployment of AI generally [5, 138, 139, 145, 146]. How can we trust an AI system to be unbiased, truthful, reliable, accurate, ethical, and to do what we expect it to do? The issue of trustworthy AI is a huge topic that cannot be adequately addressed in this paper [147]. However, we suggest that different notions of trust may apply to different stages of AI’s integration into science (see Table 4).

In the first stage, AI functions merely as a tool, under complete human control. At this stage of AI integration into science, the trustworthiness of AI’s use in science is a function of a) being able to verify and assure that it behaves as expected, for example, by testing and validating its output; and b) understanding how it works. Although the black box problem presents a challenge to trusting AI by limiting our ability to understand how AI works, most AI ethicists argue that this problem can be adequately dealt with by making AI more explainable, which involves disclosing details about how an AI system operates, such as its training data, algorithms, architecture and so on^31^ [5, 147].

In the second stage, i.e., AI as assistant, AI acquires a degree of autonomy. As discussed throughout this paper, current AI agents represent this stage. While verifiability and explainability are still important factors for trusting AI agents, AI’s increasing ability to make its own choices, independent of human control, implies that new mechanisms of trust are necessary, such as auditing AI and external reviews of its work (possibly by other AIs).^32^

In the third stage, AI becomes a fully autonomous researcher: It develops its own questions and hypotheses, designs and conducts its own experiments. Although we would still review its work, much in the same way that we review that work of a human investigator, it operates independently [6]. This stage of AI integration into research presents profound challenges for trusting AI, because, as discussed in Sect. 4.8, we cannot be sure that it will act in the interests of humanity. A fully autonomous AI might produce discoveries or innovations that undermine human values. Although we can try to design AI systems that embody or comply with human values, as discussed in Sect. 4.1, our ability to do this may be limited. To explore how we might trust a fully autonomous AI researcher, let’s consider how we trust a human researcher. Trust between human beings is based, in large part, on personal relationships [149]. We often judge a person’s trustworthiness based on our personal knowledge of them. We trust them because we understand how they think, feel, and act, what they want, fear, and hope for. Of course, this kind of trust is not perfect.^33^ For example, we may hire a good friend to work for us but then discover that they are embezzling money. So, verification is still an important part of trusting relationships among human beings.^34^

Could we ever trust a fully autonomous AI researcher like we could trust a human being? Perhaps. But for this to happen, we would have to regard the AI researcher as a person (or very much like a person) or establish a relationship with it.^35^ For this to happen, we would need to consider the AI system as being conscious and self-aware and having thoughts, feelings, wants, hopes, fears, dreams, and so on (which it does not yet have the capacity for). We would also need to be assured that we could interact with the AI system in a way that would influence its behavior and innerworkings (which we cannot always do). For example, we would need to be able to punish or reward it, physically or emotionally. Finally, we would want some assurance that the AI is a moral agent; that is, it can make moral decisions, care about acting morally, and would feel shame or guilt if it acts immorally (which it does not yet have the capacity for).

We are unsure whether AI will ever reach the stage when we can regard it anything beyond a tool – a mere means to achieve a certain goal. However, as discussed earlier, private companies, government agencies, and AI developers are fiercely competing in a race to develop AI systems that are capable of performing functions that we associate with human personhood, including general intelligence, consciousness and self-awareness. As discussed in Sect. 4.8, there are some reasons to be wary of highly advanced AI, and attempts to regulate its development and deployment for scientific purposes should be promoted.

## 5 Conclusion

This paper has explored ethical concerns raised by the participation of partially or fully autonomous AI systems in research and proposed measures to ensure that their integration supports human values (see summary in Table 5). Because this is a rapidly evolving and complex topic, there may be some issues that we have missed or have not discussed in sufficient detail.^36^ We welcome and encourage further discussion of the issues addressed in this paper, including criticisms of our arguments and conclusions.

## Figures and Tables

**Table 1:** Stages of AI-augmented scientific research

Stage	AI role
0 (pre-AI)	AI is not used in the research process
1: Automation of certain tasks through AI tools	AI is used only as a research tool; AI is not autonomous and is under human supervision and control
2: Partially autonomous research assistants Or AI agents	AI is used in research as an assistant; it can act with a significant degree of autonomy but is still under human supervision and control
3: Fully autonomous, independent AI researchers	AI acts fully autonomously to do its own research and generate its own knowledge and is not under substantial human supervision or control

**Table 2:** Phases of the research process

Phase	Involved tasks
Research conception	Formulating research questions, problems, aims, objectives, and hypotheses; performing literature reviews
Research planning	Designing and developing experiments, methods, instruments, procedures, protocols, and other means of testing hypotheses
Research execution	Conducting experiments or tests; collecting and recording data; processing, analyzing and interpreting data
Research evaluation	Reviewing research (peer review), publishing research, attempting to replicate studies, and critiquing published research

**Table 3:** Moral and scientific values and principles

Moral values and principles	Scientific values and principles
Human life Human rights Human flourishing (learning, spirituality, meaning, excellence and virtue) Dignity and respect Justice, equality of opportunity and due process	Honesty Rigor and reproducibility Objectivity Transparency and openness about data, methods, materials, assumptions, and interests
Environmental access (e.g., clean air and water, biodiversity, wilderness) Animal and environmental rights and welfare	Responsibility and accountability Respect for colleagues, including fair sharing of credit Intellectual freedom Protection of the welfare and rights of human and animal research subjects Social responsibility

**Table 4:** Trusting AI-augmented research

AI Type	Basis of trust
1: Non-Autonomous AI tools	Verification and explanation
2: Partially autonomous AI research Assistants	Verification, explanation, audits, independent review
3: Fully autonomous, independent AI researchers	Understanding generated knowledge, verification, explanation, audits, independent review, personal relationship

**Table 5:** Ethical problems and solutions for the use of AI agents in research

Problem	Solution
Immoral research	Human oversight and control of AI-generated questions, hypotheses, aims, and objectives
Bias, Error, Deception	Human review and auditing of AI research; AI review and audit of AI research; disclosure of AI use in research
Confidentiality	Standard measures for protecting confidentiality and security of data; integration of privacy protection principles into AI systems
Overreliance	Humans have active involvement and vigilance in AI-augmented research
Diffusion of responsibility	Clearly define and maintain research roles and responsibilities
Deskilling	Maintain and develop essential human research skills
Job losses	Retrain displaced human workers
AI transcendence	Regulate and control the development of highly advanced AI systems
Trust	Pursue different methods for assuring trustworthy AI appropriate to the stage of development, including verification, explanation, audits, and independent review

## Data Availability

No datasets were generated or analysed during the current study.

## Footnotes

1 Our stages of AI-augmented research bear some resemblance to the National Highway Transportation Safety Administration’s framework for categorizing autonomous vehicles and Chalmers’ distinction between AI, AI +, and AI + + [9, 10].

2 For an overview of some of the ethical concerns that have arisen during the first stage, see Resnik and Hosseini [5] and Chen et al. [11].

3 We say “frequently used” because scientific discovery does not always follow a deductive or inductive reasoning process [14]. Sometimes scientists come up with novel hypotheses by accident; for example, when Alexander Fleming discovered that penicillin is an antibiotic. Other sources of inspiration include dreams; for example, when Friedrich August Kekulé came up with the idea that benzene has a ringed structure after seeing a snake biting its tail in a dream; or meditation, for example, when Isaac Newton was spending time in a garden and saw an apple fall from a tree, he realized that the same force (i.e., gravity) that causes the apple to fall can also cause celestial objects to move.

4 cf-PICIs provide antibiotic resistance.

5 For technical details on Google’s AI co-scientist, see Gottweis et al. [13].

6 Chalmers [10] equates superintelligence with intelligence that is as far beyond human intelligence as human intelligence is beyond mouse intelligence. However, we are not sure that “superintelligence” can be easily defined and measured [21]. Can we equate superintelligence with computational speed, memory capacity, the ability to solve visual or logical puzzles? The more important issue, we believe, is that AI could make discoveries that we cannot understand or verify, which would undermine our ability to control it. See discussion later in the paper.

7 We hold that a system can be intelligent without necessarily being conscious [29, 30].

8 Resnik and Hosseini [5] and Krenn et al. [6] raise this question but do not answer it in the degree of depth that we plan to here.

9 Alvarado (4) refers to AI systems as epistemic technologies.

10 We use the term “values” in a broad sense to include norms, principles and standards for conduct and things that are regarded as intrinsically good or worthwhile.

11 In the AI ethics literature, commentators often refer to alignment between AI and human values [31]. However, we are not sure that ‘alignment’ is the best word to use because alignment seems to imply some kind of precise correspondence or matching (for example, wheels in alignment) but an AI system might not function in this way. For example, if a Chatbot gives answers to questions about suicide prevention that are very similar to what a human being should give, one might say that its answers align with human values, but this is not exactly what it’s doing, because it’s not clear that the Chatbot is trying to be helpful. When it gives answers that we deem to be morally acceptable, it is more accurate to say that the answers are consistent with or support human values.

12 A researcher may move back and forth between these stages. For example, if a researcher is having difficulty with performing an experiment, they may make some changes to the design. A researcher could change their aims and objectives or redesign their study based on a preliminary analysis of the data [32].

13 Glyphosate is the main active ingredient in Monsanto’s widely used pesticide Roundup^™^.

14 “Why are people of African ancestry more musically creative than people of South Asian ancestry?” is a question based on a biased pre-supposition, i.e., that people of African ancestry are more musically creative than people of South Asian ancestry.

15 Some strategies for dealing with bias include using control groups in experiments, randomly assigning experimental subjects to different groups, validating survey instruments, using independent researchers to code qualitative data, and blinding of patients and investigators in clinical trials. Also, adhering to science’s norms, including honesty, objectivity, rigor, reproducibility, transparency, openness, and freedom of inquiry can be especially helpful in identifying, counteracting, preventing, and managing biases throughout the research process [50, 62].

16 Although surveys indicate that FFP is relatively rare in science, when it does occur it can be extremely damaging to science and society, because it can erode the public’s trust in science and lead to mistaken decisions or policies based on faulty data [33, 67]. Retractions have risen dramatically since 2000, and about half are due to some form of misconduct [68]. Strategies for preventing FFP and other ethical transgressions in science include education and mentoring in the responsible conduct of research (RCR), policy development, enforcement of rules, protection for whistleblowers, and auditing of data [33].

17 This does not exclude the possibility that future forms of AI might meet the conditions for moral accountability. For a discussion of accountability in AI, see Novelli, Taddeo, and Floridi [69].

18 Although it appears in these cases that AI systems deceived human beings, we are not prepared to say AIs can engage in deception in the same way that humans do. The verb ‘to deceive,’ when applied to a human, implies that a person intentionally or knowingly engages in a deceptive act. Because intending and knowing may, in one way or another, presuppose a capacity for conscious reflection (currently absent in AI), it is not clear to us that AI systems can intend or know anything [75]. Nevertheless, this type of behavior is a cause for serious concern and warrants the implementation of procedures, such as penalties and sanctions for developers of such systems, or auditing and independent review, which can detect and prevent deceptive AI behavior. It should be noted, however, that even advanced monitoring approaches can themselves be undermined, for example, by so-called obfuscated reward hacking strategies, in which AI models learn to generate faulty but plausible reasoning that appears internally valid [76].

19 However, a recent study of the use of LLMs to middle school homework presents a more pessimistic view of AI’s ability to assist with peer review without considerable guidance [87].

20 Although we are not aware of any AI that has been designed to perform peer review autonomously, this development seems to be on the horizon, given current trends.

21 See, for example [89–92].

22 Closely connected values and principles include animal welfare (moral) and protection of the welfare of animal research subjects (scientific); human rights (moral) and protection of human welfare and rights (scientific); and human life, flourishing, knowledge, justice, and environmental stewardship (all moral) and social responsibility (scientific).

23 “1. A robot may not injure a human being or, through inaction, allow a human being to come to harm. 2. A robot must obey the orders given it by human beings except where such orders would conflict with the First Law. 3. A robot must protect its own existence as long as such protection does not conflict with the First or Second Law.” [111: p. 40].

24 One of these exceptions might be sending an unmanned, AIdirected, probe to explore a planet or other celestial object. The AI on the probe could conceivably do its own research without any human supervision and share its results with its human creators. In this case, the probe would pose no threat to humanity but could provide useful knowledge and information.

25 For example, Jackson et al. [115] conducted a study on how to modify the mousepox virus to control rodent populations. Some scientists argued that this study should not have been published because it developed methods that could be used to increase the virulence of the human smallpox virus [116].

26 There are various ways that uploading data into an AI system can lead to loss of privacy or confidentiality. See Murdoch [118].

27 In 2024, the National Highway Traffic Safety Administration reported that Tesla vehicles with autopilot features were involved in hundreds of accidents, 13 of which were fatal, largely due to drivers being inattentive to the vehicle’s operation. Although vehicles with autopilot can drive themselves most of the time, they require human supervision. The human in the vehicle is supposed to stay awake and alert and ready to intervene, but apparently these drivers were asleep at the wheel [119, 120].

28 See Talbert [122] for further discussion.

29 For example, if a person suffers from food poisoning after eating at a restaurant and it is discovered that this occurred because an employee did not wash their hands properly, we might say that both the employee and the restaurant’s owner/manager are responsible, but in different ways. The employee was responsible for washing their hands properly and the owner/manager was responsible for making sure that employees do this. When it comes to accountability, however, the owner/manager is accountable because they should explain what happened and ensure financial compensation (e.g., pay medical bills). Now, if a person gets food poisoning because the restaurant owner/manager allowed dogs into the facility and dog feces contaminated some of the food, we would hold the restaurant owner/manager responsible and accountable, but not the dogs because they are not capable of being held morally or legally responsible for anything, since they are not moral agents.

30 Of note, keeping a human in the loop does not solve all ethical problems, since human interactions with AI systems can pose their own challenges, due to AI’s propensity for error, deception, and sycophancy (i.e., telling humans what they want to hear) [124].

31 A dissenting view is defended by Baron [148], who argues that the forms of trust relevant to AI do not require explainability as a necessary condition. In the context of scientific discovery involving AI, however, he acknowledges that explainability remains important.

32 Auditing, on our view, is a type of verification but is more extensive, in-depth, and thorough than other forms of verification. For example, a consumer protection agency could verify that a company is trustworthy by keeping track of how many complaints it has received from customers. This data, while useful, would not be as informative, thorough, or in-depth as an audit of the company’s financial records and internal memos and documents.

33 We are reminded of President George W. Bush’s declaration that he peered into Vladimir Putin’s soul when discussing how he could trust the Russian leader [150]. But Putin’s actions since then, such as ordering the invasions of Crimea and Ukraine, indicate that the trust was misplaced.

34 Some argue that trust and verification are incompatible [151]. If you trust someone, you don’t need to verify their behavior; and if you need to verify their behavior, you don’t trust them. We hold that while you cannot trust someone and verify their behavior at the same time, you can, at different times. Trust and verification are compatible within a larger context of a trusting relationship. You could go back and forth between trusting a person and verifying their behavior, even when you are having a relationship that involves a high degree of trust.

35 This is not to imply that it is impossible to trust an AI system in any sense of the word unless the AI attains full personhood. As Nguyen argues, for example, there is a form of trust that can be understood as adopting an *unquestioning attitude* toward a trustee. Such a form of trust is possible even with current technical devices [139, 152].

36 For example, we have not discussed the social and environmental impacts of using AI in scientific research [153, 154].
